# Understanding Neighborhood Environment Related to Hong Kong Children’s Physical Activity: A Qualitative Study Using Nominal Group Technique

**DOI:** 10.1371/journal.pone.0106578

**Published:** 2014-09-04

**Authors:** Gang He, Ester Cerin, Wendy Y. Huang, Stephen H. Wong

**Affiliations:** 1 Department of Sports Science and Physical Education, The Chinese University of Hong Kong, Hong Kong, China; 2 School of Exercise and Nutrition Sciences, Deakin University, Victoria, Australia; 3 Institute of Human Performance, The University of Hong Kong, Hong Kong, China; 4 Department of Physical Education, Hong Kong Baptist University, Hong Kong, China; Arizona State University, United States of America

## Abstract

**Background:**

Relationships between the neighborhood environment and children’s physical activity have been well documented in Western countries but are less investigated in ultra-dense Asian cities. The aim of this study was to identify the environmental facilitators and barriers of physical activity behaviors among Hong Kong Chinese children using nominal group technique.

**Methods:**

Five nominal groups were conducted among 34 children aged 10–11 years from four types of neighborhoods varying in socio-economic status and walkability in Hong Kong. Environmental factors were generated by children in response to the question “What neighborhood environments do you think would increase or decrease your willingness to do physical activity?” Factors were prioritized in order of their importance to children’s physical activity.

**Results:**

Sixteen unique environmental factors, which were perceived as the most important to children’s physical activity, were identified. Factors perceived as physical activity-facilitators included “Sufficient lighting”, “Bridge or tunnel”, “Few cars on roads”, “Convenient transportation”, “Subway station”, “Recreation grounds”, “Shopping malls with air conditioning”, “Fresh air”, “Interesting animals”, and “Perfume shop”. Factors perceived as physical activity-barriers included “People who make me feel unsafe”, “Crimes nearby”, “Afraid of being taken or hurt at night”, “Hard to find toilet in shopping mall”, “Too much noise”, and “Too many people in recreation grounds”.

**Conclusions:**

Specific physical activity-related environmental facilitators and barriers, which are unique in an ultra-dense city, were identified by Hong Kong children. These initial findings can inform future examinations of the physical activity-environment relationship among children in Hong Kong and similar Asian cities.

## Introduction

Physical inactivity is now identified as the fourth leading risk factor for global mortality [1]. Participation in regular physical activity (PA) has been demonstrated to reduce the risk of high cholesterol, depression, loss of bone density, high blood pressure, obesity and metabolic syndrome among school-aged children [2]. However, evidence indicates that children are a population at high risk of physical inactivity. Accelerometry-based estimates of PA showed that only 42% of children and around 8% of adolescents in the United States met the recommendation of 60 minutes of moderate-to-vigorous PA (MVPA) per day [3]. In Hong Kong, self-reported data indicated that primary school children accrued an average of less than 55 minutes MVPA per day, and only 66% of children participated in PA after school [4]. The Leisure and Cultural Services Department of Hong Kong recently reported that only 8.3% of children and 8.4% of adolescents in Hong Kong met the recommendations of at least 60 minutes of MVPA per day [5]. These findings emphasize the need for the identification of modifiable factors impacting on children’s PA that can inform the development of effective PA promotion strategies.

According to social ecological models of health behavior [6], PA is influenced by a large number of individual, social, physical environmental and policy factors at multiple levels. Among these factors, attributes of the neighborhood physical environment have been shown to be more consistently related to children’s and adolescents’ PA than individual and social environmental factors [7]. That being the case, exploring attributes of the neighborhood environment could be particularly relevant to children’s PA. Information regarding neighborhood environments relevant to children’s PA can be derived from subjective (e.g. perception of the environment) and objective measures (e.g. audit, Geographic Information Systems; GIS) [8]. Some studies have shown that, relative to objective measures, perceptions of the environment tend to be more strongly associated with PA behaviors in both children [9] and adults [10]. Furthermore, the relationship between the objective environment and PA may be mediated by perceptions of the environment [11]. However, subjective measures of children’s neighborhood environments come predominantly from parental proxy-reports [12], [13] rather than from children’s own perceptions. Because children have unique understanding of their experiences [14], differences might exist between information collected from parents and that obtained from children. This might obscure the relationships of neighborhood environmental attributes with children’s PA. For instance, Timperio and colleagues have found that parents perceived traffic safety and public transport as correlates of their children’s walking or cycling behavior, instead, sports grounds was the correlate identified from the children’s own perspective [15]. Therefore, the study of environmental characteristics from children’s own perspectives is of particular importance for enhancing our understanding of neighborhood environmental attributes predictive of children’s PA.

Existing studies that examined the relationship between children’s perceptions of the environment and their PA were mainly conducted in Western countries [16]. Because environmental factors related to PA might be context specific, investigation of the environmental correlates needs to be conducted separately in different regions and countries. However, to our knowledge, no study has been conducted to examine the environments that are relevant to PA of Chinese Hong Kong children so far. An efficient way to investigate these issues might start from identifying environmental attributes that are important to the children using qualitative methods before a large sample survey is conducted. Being an exploratory qualitative technique, nominal group technique (NGT) has been shown to be an effective and efficient qualitative method for idea generation and decision-making [17]. NGT has several advantages: (i) it applies a highly structured process and is less time consuming than other qualitative techniques; (ii) it balances levels of participation among individuals and avoids dominant personalities and focusing effects, when compared to other group processes [18]; (iii) it is more productive in terms of idea generation relevant to other qualitative methods, e.g., brainstorming [19]; (iv) Results of NGT are lists of prioritized statements that can be easily turned into questionnaire items. Furthermore, NGT has been successfully applied in health-care research among children [20]–[22]. Therefore, the purpose of this study was to identify children’s perceived environmental facilitators and barriers to engaging in PA using NGT.

## Methods

### Ethics Statement

The study was approved by the Survey and Behavioral Research Ethics Committee of The Chinese University of Hong Kong. Consent forms were obtained from the participants and parents.

### Participants

Participants were 34 children aged 10–11 years, recruited from three primary schools and living in four types of neighborhoods varying in socio-economic status (SES) and ‘walkability’, the urban form attributes that might impact travel and activity patterns [23].

Information on median monthly household income and number of households of all TPUs (Tertiary Planning Units, the smallest census-based geographic unit used in Hong Kong; n = 287) was based on 2006 census data, retrieved from the Census and Statistics Department of Hong Kong. The land area of each TPU was obtained from the Hong Kong Planning Department. Household income was used as a measure of the SES of each TPU; household density was calculated as *number of households/land area* as an indicator of TPU walkability. TPUs with high (upper quartile) or low (lower quartile) household income were, respectively, identified as high- or low SES TPUs; TPUs with high (7^th^ to 10^th^ deciles) or low (1^st^ to 4^th^ deciles) household density were identified as high- or low walkability TPUs [24]. Four categories of TPU were therefore formed; high SES/high walkability (HSES/HW), high SES/low walkability (HSES/LW), low SES/high walkability (LSES/HW), and low SES/low walkability (LSES/LW). This recruitment strategy has been previously used in research among adolescents for maximizing heterogeneity in PA environments when studying potential associations with PA [25]. A similar approach was also adopted in study among children for investigating environment-PA relation [26].

Letters of invitation, with a brief introduction of the study, were sent to 23 randomly selected primary schools located in the four categories of TPUs. Three schools from HSES/HW, LSES/HW, and HSES/LW TPUs consented to participate in this study. In Hong Kong, TPUs usually consist of people of diverse SES backgrounds. The median household incomes of each residential building near the participating school in HSES/LW TPU were obtained from Centamap (www.centamap.com) to identify residential buildings that were of either high (e.g. detached houses) or low SES (e.g. village houses). The same procedure was adopted to identify eligible residential buildings near participating schools in HSES/HW (e.g. private estates) and LSES/HW (e.g. public renting houses) TPUs. A list of eligible residential buildings was given to teachers at each school to recruit 5^th^ grade and 6^th^ grade children living in those buildings. In total, 34 children aged 10–11 years, who lived in four types of neighborhood varying in SES and walkability, were successfully recruited. Because it is recommended that NGT groups consist of five to eight participants [17], children were divided into five groups (six to eight children per group) according to their SES and neighborhood types.

### Procedure

A highly structured procedure was used to guide the process of each NGT group [17], [18]. This procedure consisted of a series of steps which were pilot-tested beforehand:

1. Introduction of NGT purpose and process to the participants
2. Asking NGT question to the participants
3. Individual silent generation of items in writing by participants
4. Listing of items on a flip chart in a round-robin fashion
5. Discussion of items listed on the flip chart to clarify the meaning of each item
6. Preliminary vote on the items to elect five most important items which are reserved for final vote
7. Discussion of the result of preliminary vote
8. Final vote to establish the rank order of the items reserved
9. Conclusion

The NGT question addressed to the participants was: “What neighborhood environments do you think would increase or decrease your willingness to do physical activity?” Explanations were given to clarify the meaning of “physical activity”, “neighborhood”, and “environments”. “Physical activity” was defined as all body movements including not only exercise (e.g., jogging, swimming, and ball game), but also lifestyle activities (e.g. walking for recreation, walking for transportation, and climbing stairs). “Neighborhood” referred to an area within 15-minutes’ walking distance from the participant’s home. Examples were given to facilitate the understanding of “environments”. A hard copy of all the explanations and examples was also given to the participants for their reference.

There were two voting processes. In the preliminary vote process, each participant was asked to choose three items that he/she personally considered the most important and to write them down, in order of priority, on a sheet; the first priority was assigned a score of three, the second priority was assigned a score of two, the third priority was assigned a score of one. Five items with the highest scores were reserved for the final vote. The number of items could, in fact, be more than five. If, for example, items notionally ranking from 4^th^ to 8^th^ had the same score, these items would all be included. The reason for reserving only high-scoring items was that these items were perceived to be relatively more important than others to the participants. In the final vote process, each participant was given a new sheet to vote for the items which were reserved during the preliminary vote in order of his or her personal priority (even if their orders were the same as in the preliminary vote). Participants were also required to assign a score of (for example) five to the first priority, four to the second priority, and so on. The highest score could vary and was equal to the number of items reserved during the preliminary vote; thus, if eight items were kept in the preliminary vote, the highest score would be eight. The order of importance of items was then established according to the score they received in the final vote process.

## Results

### Characteristics of NGT Groups

Thirty-four primary school children from fifth and sixth grades took part in this study (Table 1). All NGT groups were conducted in classrooms during school time and completed within one hour. In response to the question “What neighborhood environments do you think would increase or decrease your willingness to do physical activity?”, a total of 117 items were generated from five NGT groups; 39 items from two HSES/HW groups, 14 items from one HSES/LW group, 35 items from one LSES/HW group, and 29 items from one LSES/HW group. Within all the items generated, a total of 30 items receiving the highest scores in the preliminary vote were reserved for further processing.

**Table 1:** Characteristic of NGT Groups.

	Neighborhood type	No. of participants	Age of participants	No. of items generated	No. of items reserved
Group 1	HSES/HW	6 (boys = 3)	10 (5^th^ grade)	21	6
Group 2	HSES/HW	6 (boys = 3)	10 (5^th^ grade)	18	5
Group 3	HSES/LW	8 (boys = 4)	11 (6^th^ grade)	14	5
Group 4	LSES/HW	6 (boys = 3)	11 (6^th^ grade)	35	8
Group 5	LSES/LW	8 (boys = 4)	11 (6^th^ grade)	29	6

### PA-related Neighborhood Environmental Factors Identified by NGT Groups

The 30 reserved items–in other words, neighborhood environmental factors that children perceived as important for their willingness to engage in PA–are listed in Table 2.

**Table 2:** Reserved Items Generated from Each NGT Group.

NGT Group	Items	Score^a^ in finalvote	Score^a^ inpreliminary vote	No. of votes^b^ inpreliminary vote
**Group 1**	Items = 6	Range = 6–36	Range = 0–18	Range = 0–6
(n = 6)	Strange people on streets^c^	25	6	2
HSES/HW	Parks	24	5	2
	Recreation grounds	24	3	1
	Sufficient lighting	20	3	2
	Shopping malls with air conditioning	18	3	1
	Bridge or tunnel	15	3	1
**Group 2**	Items = 5	Range = 6–36	Range = 0–18	Range = 0–6
(n = 6)	Dangerous people on the bridge^c^	27	10	4
HSES/HW	Fresh air	20	5	2
	Too much noise* c	18	5	3
	Subway station	14	3	1
	Hard to find toilet in shopping mall^c^	11	3	1
**Group 3**	Items = 5	Range = 8–40	Range = 0–24	Range = 0–8
(n = 8)	Recreation grounds	31	17	6
HSES/LW	Crime at night^c^	31	9	4
	Inconvenient transportation^c^	24	7	3
	Too many people in recreation grounds^c^	19	6	5
	Fresh air	15	3	2
**Group 4**	Items = 8	Range = 6–48	Range = 0–18	Range = 0–6
(n = 6)	Dangerous people on streets^c^	45	7	3
LSES/HW	Insane people on streets^c^	39	4	3
	Addicts on streets^c^	35	3	1
	Strangers in parks^c^	30	3	1
	Afraid of being taken or hurt at night^c^	23	3	1
	Recreation grounds	19	3	1
	Convenient transportation	16	3	2
	Perfume shop	9	3	1
**Group 5**	Items = 6	Range = 8–48	Range = 0–24	Range = 0–8
(n = 8)	Crimes nearby^c^	41	11	5
LSES/LW	Strange people on streets^c^	36	6	2
	Public order is good	27	5	2
	Interesting animals	24	5	2
	Fewer cars on roads	21	3	1
	Fresh air	19	4	2

a Score of an item in two voting processes was calculated by summing up the individual score across all participants in a NGT group. It indicated the relative importance of an item within a NGT group. In the preliminary vote process, as an item would receive a score ranging from 0 (not chosen as a priority) to 3 (chosen as the first priority) from each participant, the theoretical range of score would be from (0*number of participants in a NGT group) to (3*number of participants in a NGT group); in the final vote process, as an item would receive a score ranging from 1 to as high as number of reserved items in a NGT group, the theoretical range of score would be from (1* number of participants in a NGT group) to (number of reserved items in a NGT group * number of participants in the same NGT group).

b Votes refer to number of times an item was selected as a priority by participants during the preliminary vote, regardless of the score assigned to it. The theoretical range of votes would be from 0 to number of participants in a NGT group.

c Factors perceived to decrease the participants’ willingness to do PA.

Items were further processed to avoid duplication: 1) Items with similar meaning generated within an NGT group were conflated into a single item. In Group 4, “Dangerous people on streets”, “Insane people on streets”, “Addicts on streets”, and “Strangers in parks” were synthesized as “People who make me feel unsafe in streets/parks”. In Group 5, “Crimes nearby” and “Public order is good” were conflated as “Crimes nearby”. 2) Items with similar meaning generated from different NGT groups were reworded to a comparable expression. “Strange people on streets” (Group 1) and “Dangerous people on the bridge” (Group 2) were reworded as “People who make me feel unsafe in streets/parks”; “Parks” (Group 1) were grouped into “Recreation grounds”; “Crimes during night” (Group 3) was reworded as “Crimes nearby” and “Inconvenient transportation” (Group 3) was reworded as “Convenient transportation”. 3) Because participants in Group 1 and Group 2 were from the same neighborhood type (HSES/HW), items generated from these two groups were combined.

Results of the NGT process are shown in Table 3. A total of 16 neighborhood environmental factors were identified and grouped according to Pikora’s framework [27], comprising six safety-related factors, three functionality-related factors, two destination-related factors, four aesthetic-related factors, and one factor that did not fall into any of the framework categories. Six of the 16 factors were perceived to decrease the participants’ willingness to engage in PA.

**Table 3:** PA-related Neighborhood Environmental Factors Identified by NGT Groups.

Category	Items	Votes from NGT Groups of fourneighborhood types				Cumulative votes^a^
		HSES/HW	HSES/LW	LSES/HW	LSES/LW	
**Safety** (crime)	People who makeme feel unsafe^b^	6	–	8	2	16
	Crimes nearby^b^	–	4	–	7	11
	Sufficient lighting	2	–	–	–	2
	Afraid of being takenor hurt at night^b^	–	–	1	–	1
**Safety** (traffic)	Bridge or tunnel	1	–	–	–	1
	Few cars on roads	1	–	–	–	1
**Functionality**	Convenient transportation	–	3	2	–	5
	Subway station	1	–	–	–	1
	Hard to find toilet inshopping mall^b^	1	–	–	–	1
**Destination**	Recreation grounds	3	6	1	–	10
	Shopping malls withair conditioning	1	–	–	–	1
**Aesthetic**	Fresh air	2	2	–	2	6
	Too much noise^b^	3	–	–	–	3
	Interesting animals	–	–	–	2	2
	Perfume shop	–	–	1	–	1
**Others**	Too many people inrecreation grounds^b^	–	5	–	–	5

a Votes added up across all NGT groups, used as an indicator of the importance of an item across all participants.

b factors perceived to decrease the participants’ willingness to do PA.

## Discussion

This study was conducted to determine neighborhood environmental characteristics that were perceived by children as factors influencing their PA. This is one of the few studies to date, if any, attempting to understand children’s perceptions of environmental facilitators and barriers to engaging in PA in the neighborhood using NGT. In total, 16 factors were identified by five NGT groups, consisting of children from four types of Hong Kong neighborhoods varying in SES and walkability.

Hong Kong, like other Asian metropolises, differs from Western cities by its extreme level of population density. For example, there are 2.39 million residential units (2011 Hong Kong Population Census, Census and Statistics Department, Hong Kong Government, 2011) living in a total of 7,600-hectare residential area (Planning Department, Hong Kong Government, 2013) in Hong Kong, i.e., the average residential density of Hong Kong is 314 residential units per hectare. In contrast, the residential density of European cities seldom exceeds 125 residential units per hectare (www.plan4sustainabletravel.org). This ultra-dense context could lead to environmental characteristics that are uncommon to Western locations but impact on children’s PA. Examples are air or noise pollution, crowdedness, a complex public transport network, and extensive indoor areas for walking [24]. Among the total of 16 environmental factors identified in this study, five factors (bridge or tunnel, shopping malls with air conditioning, fresh air, too much noise, and too many people in recreation grounds), according to previous research [24], are deemed to represent environmental characteristics particularly relevant to Hong Kong or similar ultra-dense East Asian cities; two factors (hard to find toilet in shopping mall and perfume shop), to our knowledge, were never addressed in literature; whereas the rest of the factors are common attributes shared by most urban environments.

As documented in the literature [28], perceived safety was a correlate frequently examined by researchers. In this study, safety was also shown to be a major concern. In fact, six out of the total sixteen factors identified in this study were related to safety, four of which were related to crime safety. Among these, “People who make me feel unsafe” and “Crimes nearby” received the most cumulative votes (16 and 11 respectively), and votes were derived from all NGT groups, indicating that crime safety was a major and common barrier to PA participation for Hong Kong children. However, studies among Chinese children failed to find an association between perceived safety variables and children’s PA [29]. A recent study conducted among Hong Kong children also indicated that children’s “worry about strangers” did not relate to their PA [30]. Existing studies concerning environment safety usually just asked about a general ‘impression’ of safety which nearly all children and parents identified as an issue. Thus, there was not much variability on this factor. Additionally, previous studies focused on overall children’s PA rather than context-specific PA (i.e., PA undertaken within the neighborhood of residence). Neighborhood safety is more likely to impact on PA performed within the neighborhood than that performed at school. Future research should investigate this issue in more depth by unpacking the meaning of ‘safety’ and measuring context-specific PA to clarify the relationship between neighborhood safety and PA in children.

As to traffic safety, two factors (“Bridge or tunnel” and “Few cars on roads”) were identified by the participants. The traffic load in Hong Kong is relatively heavy. Data from Hong Kong Government indicate that there are about 290 vehicles for every kilometer of road in Hong Kong (Transport Department, Hong Kong Government, 2013). Therefore, traffic load might be a serious concern with respect to children’s walking or playing on the streets. A qualitative study identified that parents perceived too many cars on the street as being a safety concern when allowing their children to play in the neighborhood [31]. However, there is empirical evidence that children whose parents perceived there was heavy traffic in local streets were more likely to commute actively [15], [32]. This might be because of parents whose children engaged in active commuting being more aware of the traffic conditions [15]. Because of heavy traffic, Hong Kong is typified by the presence of bridges or tunnels which help pedestrians to safely cross busy roads. Being a factor specific to Hong Kong, this environmental aspect was not investigated in previous studies.

A total of three functional factors were identified in this study. Two of these (“Convenient transportation” and “Subway station”) concerned public transport. The public transport system in Hong Kong is highly developed, comprising railways, trams, buses, minibuses, taxis and ferries. Participants mentioned that the presence of Mass Transit Railway (MTR) stations or bus stops near their home encouraged them to walk to these destinations for transportation purposes. This is in line with findings from a previous study according to which limited public transport had a negative impact on walking behavior in girls [15]. However, when examining PA as a whole, perceived “convenience” bore no relation with children’s self-reported overall PA [29]. This suggests that future studies examining the relation between transportation convenience and PA need to be behavior-specific (e.g. walking for transportation vs. overall PA), because transportation-related environmental factors are more likely to impact walking behavior or commuting mode than overall PA. Moreover, context of PA (e.g., PA on school days vs. PA on weekends) should also be considered. In fact, transportation convenience is more relevant to children’s walking behavior when they have free choice (e.g., on weekends). Children mentioned “Hard to find toilet in shopping mall” as a barrier to their PA in this study, which is a previously undocumented finding. Given that in Hong Kong toilets are commonly available in shopping malls, this could indicate the lack of clear signage.

Among the two factors identified in the destination category, “Recreation grounds (including parks)” was mentioned as an important destination by participants from three neighborhood types (HSES/HW, HSES/LW, and LSES/HW). Although research among Hong Kong children did not find a significant association between availability of sports facilities and children’s PA [30], many studies showed consistent positive results. Timperio and colleagues [15] indicated that children who perceived no parks or sports grounds near home showed a lower likelihood of walking or cycling. Hume et al. [33] provided a list of 15 destinations (seven of which were related to recreation grounds) to 280 ten-year-old children and asked them to report the number of destinations inside their neighborhood. Results showed that the number reported was positively associated with boys’ walking behavior. Huang et al. [29] conducted a study among children in Taiwan and found that perceived accessibility to 22 recreation grounds was positively related to children’s PA. “Shopping malls with air conditioning”, another factor identified by the participants, could also be considered as space for children to play, because the participants mentioned that “it is comfortable to play in shopping malls with air conditioning, and is safe”. In the hot and humid environment of Hong Kong, indoor areas like shopping malls do provide a comfortable space for walking [24].

A total of four factors were identified in the category of aesthetics. “Fresh air” and “Too much noise” (pollution-related factors) were identified as major concerns. These have also been identified as important characteristics shared by East Asian cities [24]. The presence of interesting animals was identified as an important characteristic by participants from LSES/LW neighborhoods. LSES/LW areas of Hong Kong are usually relatively low-density villages, where animals can be frequently seen during walks. “Perfume shops” were proposed as aesthetically pleasing features making the environment more enjoyable. None of the four aesthetic factors identified in this study had been previously investigated. Previous studies mainly focused on the appearance of neighborhood built environments [29], [33]. No significant relationships were found between perceived aesthetics and children’s overall PA [29], although a pleasant-looking neighborhood was positively associated with girls’ walking frequency [33]. “Too many people in recreation grounds”, the single factor that could not be grouped into the above categories, was also shown to be an attribute common among East Asian ultra-dense cities [24]. However, the impact of this factor on children’s participation in PA needs to be further examined.

Overall, this study provides preliminary findings of how children in a dense urban context perceived environmental factors that may influence their PA. These findings are in line with Pikora’s framework according to which environmental features such as safety, functionality, destinations, and aesthetic influence PA behaviors [27]. As to specific environmental factors generated in this study, crime-related safety (e.g. “People who make me feel unsafe”, “Crimes nearby”, “Sufficient lighting”, and “Afraid of being taken or hurt at night”), traffic speed/volume (e.g. “Few cars on roads”), and recreation facilities (e.g. “Recreation grounds”) have been extensively investigated in Western countries despite their inconsistent associations with children’s PA [16]. The impact of public transportation (corresponding to the themes “Convenient transportation” and “Subway station” in the present study) on children’s PA has been less extensively examined in Western countries probably due to the lower prevalence of public transportation in these countries [34]. Interesting things to look at (corresponding to the theme “Interesting animals” in the current study) has been included as one of the items gauging perceived aesthetics in U.S. children [35], [36]. Overall perceived aesthetics has been found to be related to children’s PA [35], [36]. Despite these common factors shared by major Western urban environments, attributes representing the compact (“Bridge or tunnel”, “Too many people in recreation grounds”), hot and humid (“Shopping malls with air conditioning”), and polluted (“Fresh air”, “Too much noise”) environment are unique to Hong Kong and similar dense cities. They have also been identified as important factors among Hong Kong adults [24]. Two novel factors, i.e. “Hard to find toilet in shopping mall” and “Perfume shop”, have not been reported before. It should be noted that all the environmental factors generated in this study were perceived to influence children’s “willingness to do PA”, their impact on or associations with children’s walking or PA behaviors should be further examined.

This study has several strengths. It is among the first of its type in attempting, using NGT, to understand how neighborhood environments might relate to children’s PA. Factors identified in this study were all proposed by Hong Kong children, instead of being adopted from existing questionnaires developed in Western countries. Therefore, these factors might be more relevant neighborhood environmental correlates of children’s PA in the Hong Kong context. Findings from this study could be used for developing or complementing study instruments in related areas. This study has also applied a stratified sampling method, whereby participants were stratified by neighborhood types. This sampling method balanced the presentation of opinions from different neighborhoods, i.e. high/low SES and high/low walkability and is consistent with international research studies that have examined associations between the environment and PA among adults [23] and youth [25].

This study has also several limitations. First, household density was used as the only indicator of walkability. However, a higher density may not always foster walking or PA. Although density is usually associated with a higher availability of destinations [37], it may also result in air or noise pollution and crowdedness which may hinder active behaviors as reported previously [24]. If possible, future studies should include more indicators (e.g., intersection density, commercial and destination density, land use patterns) to make the measurement of walkability more accurate [38]. Second, because of the limitation on the time allowed for each NGT group and the cognitive ability of the participants, “physical activity” in this study was not divided into specific types (e.g., walking for transportation, walking for recreation, and structured exercise). So this study was not able to shed light on the environmental factors perceived to be related to specific types of activity.

## Conclusion

A total of 16 neighborhood environmental factors were identified as either facilitators or barriers to PA by Hong Kong children. Future research that examines the association between environmental factors identified in this study and children’s specific types of PA in Hong Kong and similar Asian cities is warranted.
